# Effects of omega-3 PUFA-enriched egg consumption on metabolic parameters in elderly adults with metabolic syndrome: study protocol for a randomized controlled trial

**DOI:** 10.3389/fnut.2026.1831128

**Published:** 2026-05-19

**Authors:** Haiyue Yang, Na Zhang

**Affiliations:** 1 Department of Nutrition and Food Hygiene, School of Public Health, Peking University, Beijing, China; 2 Laboratory of Toxicological Research and Risk Assessment for Food Safety, Peking University, Beijing, China

**Keywords:** elderly adults, lipid metabolism, metabolic syndrome, omega-3 PUFA-enriched eggs, rct

## Abstract

**Background:**

Metabolic syndrome (MetS) affects approximately one-third of community-dwelling older adults (≥65 years) and is characterized by concurrent dysregulation of glucose and lipid metabolism alongside chronic low-grade inflammation. Poor long-term adherence to conventional omega-3 supplementation has prompted interest in sustainable, food-based delivery systems. Omega-3 polyunsaturated fatty acid (PUFA)-enriched eggs—produced through dietary modification of hen feed to enhance eicosapentaenoic acid (EPA) and docosahexaenoic acid (DHA) content-represent a palatable and easily incorporated dietary vehicle for sustained intervention.

**Methods:**

This is a three-arm, parallel-group randomized controlled trial that will enroll 180 community-dwelling adults aged ≥ 65 years with MetS. Participants will be randomly allocated (1:1:1) to receive either omega-3 PUFA-enriched eggs, regular eggs, or no egg intervention (usual diet) for 12 weeks. Those assigned to the egg intervention groups will consume one boiled egg daily, five days per week. The control group will maintain their habitual dietary patterns without additional egg consumption. Biological samples (blood, urine, and stool) will be collected at baseline and weeks 4, 8, and 12. Plasma concentrations of omega-3 fatty acids, lipid metabolism indicators,inflammation and oxidative stress markers will be measured. Exploratory outcomes include comprehensive multi-omics profiling, encompassing metabolomics and gut microbiome will be tested.

**Results:**

The primary outcome will be the longitudinal changes in fasting triglycerides (TG) measured at Weeks 0, 4, 8, and 12, with comparisons between the omega-3 PUFA-enriched eggs group and the regular eggs control group. Secondary outcomes will include glucose levels, insulin concentrations, lipid profiles, as well as biomarkers of inflammation and oxidative stress. Integrative multi-omics analyses will be conducted to explore the pathways linking omega-3 PUFA-enriched eggs consumption to alterations in metabolomic features and gut microbiota.

**Conclusions:**

This trial will aim to assess the efficacy, feasibility, and mechanisms of omega-3 PUFA-enriched eggs in elderly adults with MetS, so as to provide insights and evidence to support scalable community-based dietary interventions.

**Trial registration:**

The protocol has been registered on the website of Chinese Clinical Trial Registry. The Identifier code is ChiCTR2600120125. The Registry date is 10 March, 2026. Registry name is “Interventional Effect of ω-3 Polyunsaturated Fatty Acid-Fortified Eggs on Elderly Patients with Metabolic Syndrome.” Registration URL is [https://www.chictr.org.cn/bin/userProject].

## 1 Introduction

Metabolic syndrome (MetS), one of the most prevalent chronic disorders worldwide, is a clinical entity characterized by central obesity, hypertension, dysglycemia, elevated triglycerides, and reduced high-density lipoprotein cholesterol (1). Its core pathophysiological characteristics include insulin resistance, disordered glucose and lipid metabolism, and concomitant low-grade chronic inflammation (2). The prevalence of metabolic syndrome (MetS) remains high among community-dwelling older adults aged 65 years and older, and is associated with elevated risks of cardiovascular disease, type 2 diabetes mellitus, and physical functional decline. Furthermore, chronic low-grade inflammation, as reflected by increased high-sensitivity C-reactive protein (hsCRP), frequently coexists with MetS and multidimensional frailty. This indicates that disordered glucose and lipid metabolism coupled with chronic inflammation may constitute a key common pathway underlying adverse clinical outcomes in older adults with MetS (3). With global aging accelerating, identifying effective, safe, and easily implementable intervention strategies for metabolic syndrome has become an urgent public health and clinical research priority.

Omega-3 polyunsaturated fatty acids (PUFAs), mainly eicosapentaenoic acid (EPA) and docosahexaenoic acid (DHA), have been extensively investigated for their potential in mitigating metabolic risk. They exert beneficial effects by regulating lipid metabolism, enhancing insulin sensitivity, and mediating strong anti-inflammatory responses (4, 5). Numerous observational studies and clinical trials have demonstrated that increased omega-3 PUFA intake is associated with improvements in individual components of metabolic syndrome. Mechanistically, omega-3 PUFAs may modulate multi-level network interactions across lipidomic, transcriptomic, inflammatory signaling, microbiomic, and metabolomic pathways, thereby affecting lipid synthesis and transport (6). Systematic reviews and meta-analyses of randomized controlled trials have consistently demonstrated that marine-derived omega-3 supplementation results in triglyceride (TG) reductions in a dose- and duration-dependent manner among individuals with metabolic syndrome. Nevertheless, the beneficial effects on high-density lipoprotein cholesterol (HDL-C), blood pressure, and fasting blood glucose remain inconsistent, largely attributable to heterogeneity across studies and limited sample sizes (7). Traditional omega-3 supplementation largely depends on fish oil capsules or increased consumption of fatty fish. EPA and DHA are primarily derived from marine sources, whereas the *in vivo* conversion efficiency of plant-derived α-linolenic acid (ALA) to EPA and DHA is limited. Furthermore, the average dietary intake of EPA and DHA is merely 0.067 g/day (0.03%E), which is far below the recommended daily intake of 0.25–2.00 g/day for omega-3 polyunsaturated fatty acids (8). Such a gap poses substantial challenges to achieving effective dietary exposure levels. Moreover, elderly individuals frequently encounter compliance barriers including taste, odor, cost, swallowing difficulties, gastrointestinal intolerance, and poor long-term adherence, which considerably restrict the broad practical application of such interventions (9–11). Therefore, the key challenge lies in identifying a sustainable, palatable dietary vehicle that can effectively integrate omega-3 interventions into daily dietary practice.

Therefore, it is critical to identify a practical dietary vehicle capable of efficiently delivering omega-3 PUFAs while supporting long-term adherence. This objective may be effectively achieved through the use of omega-3 PUFA-enriched eggs, which are produced by adjusting the diet of laying hens to elevate the content of EPA and DHA (12). Eggs are widely accepted, convenient to consume, and represent a staple in daily diets, rendering them an ideal “invisible” carrier for omega-3 polyunsaturated fatty acids. This strategy is highly consistent with the dietary habits of older adults, supports standardized production and monitoring, and may enhance the feasibility and long-term adherence of interventions. Recent studies have showed that intake of omega-3 PUFA-enriched eggs effectively elevates plasma omega-3 fatty acid concentrations and improves lipid profiles in healthy adults (13–15). In addition, a double-blind randomized controlled trial reported that three months of continuous consumption of omega-3 PUFA-enriched eggs (also fortified with omega-5 and omega-7 fatty acids) significantly reduced waist circumference among individuals at risk of metabolic syndrome, suggesting potential benefits against central obesity—a core phenotypic feature of MetS (16).

Nevertheless, current evidence remains limited in several aspects. First, most existing studies on omega-3–enriched eggs have focused on general adult populations or individuals at metabolic risk, with a lack of high-quality evidence among elderly patients with confirmed metabolic syndrome. This makes it difficult to draw reliable conclusions regarding the true intervention effect in this clinically important subgroup (16). Second, some studies focus on conventional clinical endpoints, including serum lipid and glucose concentrations, yet mechanistic research remains insufficient. While the metabolic benefits of omega-3 fatty acids have been associated with lipid remodeling, inflammatory regulation, and gut microbiota alterations, human trial findings remain inconsistent, accompanied by marked interindividual variability. A recent large-scale double-blind randomized controlled trial among patients with type 2 diabetes and hypertriglyceridemia demonstrated that fish oil supplementation significantly lowered triglyceride levels and remodeled the serum lipidome, while producing only minimal effects on overall gut microbiota composition. Notably, baseline gut microbiota profiles strongly predicted triglyceride-lowering responses, with partial mediation by specific lipid metabolites. These observations indicate that the microbiota–metabolite–clinical phenotype regulatory pathway warrants further validation in populations with metabolic syndrome (17). Finally, the interactions between omega-3 polyunsaturated fatty acids and gut microbiota, as well as their metabolites such as short-chain fatty acids, have emerged as a key mechanistic framework underlying the health benefits of omega-3 supplementation (18). However, systematic evaluations of omega-3 PUFA-enriched egg interventions focusing on gut microbiota and host metabolome regulation in elderly patients with MetS remain scarce. Therefore, a rigorously designed and practically feasible clinical trial is urgently needed to bridge the gap between clinical evidence and mechanistic insights.

This study primarily hypothesized that regular consumption of omega-3 PUFA-enriched eggs by elderly patients with MetS could improve glucose and lipid metabolism and chronic inflammation compared to those consuming regular eggs (control intervention group) or receiving no intervention (blank control group). The underlying mechanisms involve remodeling of lipid metabolic pathways, alterations in gut microbial composition, and changes in key metabolite profiles. This study aimed to investigate the impact of omega-3 PUFA enriched eggs on markers of glycemic and lipid metabolism (e.g., TG, HDL-C, fasting blood glucose, and insulin resistance) and inflammatory indicators in elderly MetS patients. It further aimed to examine changes in gut microbial composition and associated clinical metabolic phenotypes following intervention. Additionally, this study sought to identify key metabolites altered by omega-3 PUFA-enriched egg consumption and establish a multi-omics framework to clarify variations in intervention effectiveness. Finally, by integrating omics data with pathway analysis, this study aimed to explore potential biological mechanisms underlying the metabolic benefits of omega-3 PUFA-enriched eggs in elderly individuals with MetS.

This study will primarily hypothesize that consumption of omega-3 PUFA-enriched eggs by elderly patients with MetS will improve glucose and lipid metabolism and chronic inflammation, compared with those who consume regular eggs or receive no intervention (blank control group). The underlying mechanisms will involve remodeling of lipid metabolic pathways, alterations in gut microbial composition, and changes in key metabolite profiles. This study will aim to investigate the impact of omega-3 PUFA-enriched eggs on markers of glycemic and lipid metabolism and inflammatory indicators in elderly MetS patients. It will further aim to examine changes in gut microbial composition and associated clinical metabolic phenotypes following the intervention. Additionally, this study will seek to identify key metabolites altered by omega-3 PUFA-enriched egg consumption and establish a multi-omics framework to clarify variations in intervention effectiveness. Finally, by integrating omics data with pathway analysis, this study will aim to explore potential biological mechanisms underlying the metabolic benefits of omega-3 PUFA-enriched eggs in elderly individuals with MetS.

## 2 Methods

### 2.1 Study hypothesis

This study hypothesizes that sustained, regular consumption of omega-3 PUFA-enriched eggs in elderly adults with MetS will improve glucose and lipid metabolism and attenuate chronic low-grade inflammation. The underlying mechanisms are postulated to involve remodeling of lipid metabolic pathways, modulation of gut microbial ecology, and alterations in key metabolite profiles.

### 2.2 Study design

This study is designed as a three-arm, parallel-group randomized controlled trial that will recruit 180 older adults with MetS. Eligible participants will be randomized equally to: (i) omega-3 PUFA-enriched egg intervention; (ii) standard egg control; or (iii) usual-diet control, with a 12-week follow-up period. Post-intervention outcomes will assess the impact of enriched eggs on MetS indicators and elucidate mechanistic pathways. This protocol is registered with the WHO International Clinical Trials Registry Platform (Table 1).

**Table 1:** Items from the world health organization international clinical trials registry platform data set.

Item	Information
**Registration number**	ChiCTR2600120125
**Date of registration**	2026-03-10
**Registration status**	Prospective registration
**Public title**	Interventional Effect of ω-3 Polyunsaturated Fatty Acid-Fortified Eggs on Elderly Patients with Metabolic Syndrome
**Scientific title**	Interventional Effect of ω-3 Polyunsaturated Fatty Acid-Fortified Eggs on Elderly Patients with Metabolic Syndrome
**Name of the ethic committee**	Institutional Review Board of Peking University
**Approved No. of ethic committee:**	IRB00001052-25192
**Date of approved by ethic** **committee**	2026-01-05
**Study type**	Interventional Study
**Study design**	Parallel, a three-arm, parallel-group randomized controlled trial
**Objectives of study**	To determine the effects of 12-week omega-3 PUFA-enriched egg consumption on glucolipid metabolism, gut microbiota composition, and systemic metabolomic profiles, and to elucidate the underlying integrative mechanisms in older adults with metabolic syndrome.
**Key inclusion and exclusion criteria**	Inclusion Criteria: Community-dwelling adults aged ≥65 years with a clinical diagnosis of metabolic syndrome. Exclusion Criteria: Medical Conditions: Severe hepatic or renal impairment, immunodeficiency disorders, active infectious diseases, or type 1 diabetes; malignant hypertension, malignancy, thyroid dysfunction (hyperthyroidism or hypothyroidism), or electrolyte disturbances; acute diabetic complications or severe chronic complications, including cardiovascular or cerebrovascular events, within the preceding month; prior bariatric surgery or recent significant weight change (unintentional or intentional loss >5% over 3 months or >10% over 6 months). Dietary Factors: adherence to specialized therapeutic diets (e.g., gluten-free for celiac disease) or any substantial dietary modification within 12 weeks prior to enrollment. Cognitive and Functional Status: severe medical or psychiatric disorders, or cognitive, visual, or hearing impairments that would preclude study compliance. Medication and Supplement Use: current use of omega-3 PUFA supplements (fish oil, omega-3 fatty acids, DHA, EPA, or ALA) or medications known to affect lipid metabolism. Concomitant medication use is systematically documented during the screening visit via a comprehensive medication questionnaire, capturing all prescription and over-the-counter drugs and supplements, including product names and duration of use. Allergies: documented egg allergy.
**Interventions**	Group1: Omega-3 PUFA-enriched eggs group Intervention: Participants will consume one omega-3 PUFA-enriched egg daily, five days per week (Tuesday through Saturday). Monday and Sunday are designated as egg-free days, during which no eggs or egg-derived products are permitted. Concurrent use of deep-sea fish oil and other omega-3 fatty acid supplements is prohibited throughout the intervention period. Group2: Regular eggs group Intervention: Participants will consume one regular egg daily, five days per week (Tuesday through Saturday). Monday and Sunday are designated as egg-free days, during which no eggs or egg-derived products are permitted. Concurrent use of deep-sea fish oil and other omega-3 fatty acid supplements is prohibited throughout the intervention period. Group3: blank control Group Intervention: Participants will maintain their habitual dietary patterns throughout the 12-week study period. Consumption of deep-sea fish oil and other omega-3 fatty acid supplements is prohibited for the duration of the trial.
**Outcomes**	Plasma concentrations of omega-3 fatty acids, lipid metabolism indicators,inflammation and oxidative stress markers; metabolomics and gut microbiome
**Collecting samples**	Blood, Urine, Fecal samples
**Recruitment state**	Not yet recruiting
**Randomization procedure**	The randomization sequence will be generated by an independent statistician (Jiaxuan Shi, Peking University School of Public Health) who is not involved in trial conduct or outcome assessment. Using simple randomization, participants will be allocated in a 1:1:1 ratio to one of three group: (i) omega-3 PUFA-enriched eggs intervention; (ii) regular eggs group; or (iii) blank control group. Randomization will be performed via the sample() function in R software (version 4.3.1). The sequential allocation list will be encrypted and stored in a secure, access-controlled repository. To ensure allocation concealment, the randomization schedule will remain inaccessible to intervention administrators, outcome assessors, and data analysts until the completion of data collection and the prespecified unblinding procedure.
**Blinding**	An open-label control with blinded intervention arms design will be adopted in this study. Of the three randomly allocated groups, only the blank control group will be unblinded and aware of their non-intervention status. The control group and the omega-3 PUFA-enriched eggs group will be administered using a blinded protocol, where participants will be informed that they will receive egg intervention but will remain blinded to the specific type (regular fresh eggs vs. omega-3 PUFA-enriched eggs). Blinded personnel will also include intervention administrators, outcome assessors, and data analysts. Throughout the entire trial, intervention administrators, outcome assessors, and data analysts will be informed only of the unique identification codes of the participants and will be unaware of the intervention types corresponding to each group. The random allocation table will be encrypted and stored in a secure, access-controlled repository by an independent third-party biostatistician, and centralized unblinding will be performed only after the trial has been completed and the data have been locked.
**Data collection and management**	Data will be collected using paper-based or electronic Case Report Forms (CRFs). The content of the CRFs will cover modules including participants' baseline characteristics, intervention implementation status, outcome indicator test results, and adverse event records. All data entries will be completed by trained researchers to ensure the authenticity, accuracy, and completeness of the data. A standardized Electronic Data Capture (EDC) system will be used for data collection and management, and the specific system to be used will be finalized prior to trial initiation.

### 2.3 Sample size calculation

This three-arm randomized controlled trial will adopt an allocation ratio of 1:1:1. The primary outcome will be the change in fasting serum triglyceride (TG) levels from baseline to the end of the intervention. The main effect to be tested will be the difference in TG level changes between the omega-3 PUFA-enriched eggs intervention group and the regular eggs group. Based on previous dietary intervention studies in populations with metabolic syndrome, the estimated standard deviation of fasting serum TG is approximately 58 mg/dL (baseline TG level: 140.9 ± 58.0 mg/dL) (19). Sample size estimation will be based on data from a prior randomized trial, which reported a 35 mg/dL difference in triglyceride (TG) levels between participants consuming omega-3 PUFA-enriched eggs (152.476 mg/dL) and those consuming regular eggs (187.333 mg/dL) (20). Accordingly, the minimal clinically meaningful difference (δ) will be set at 35 mg/dL. Assuming a two-sided significance level of 0.05 and 80% statistical power (1–β = 0.80), the required sample size will be computed using the standard formula for comparing means between independent groups:


$$\begin{array}{l} N=\frac{2{\left(Z_{1-\frac{\alpha }{2}}+Z_{1-\beta }\right)}^{2}}{\delta^{2}} \end{array}$$

Where Z_1−α/2_ = 1.96, Z_1−β_ = 0.84, σ = 58 mg/dL, and δ = 35 mg/dL. Consequently, to meet the statistical power requirements for the primary comparison, approximately 44 participants per group will be needed. Considering a potential dropout rate of approximately 25% in 12-week dietary intervention studies among elderly populations (21), the sample size will increase accordingly:


$$\begin{array}{l} n=\frac{44}{1-0.25}\approx 59 \end{array}$$

Therefore, the final target sample size will be set at 60 participants per group, yielding a total of 180 participants across the three arms.

### 2.4 Study participants

Participants will be recruited from Guangyang District, Langfang City, Hebei Province, using purposive and convenience sampling methods. Recruitment strategies will include community health clinic screenings, health education sessions, and telephone invitations.

Inclusion Criteria: Community-dwelling adults aged ≥ 65 years with a clinical diagnosis of MetS will be included. MetS will be defined as meeting at least three of the following five criteria: (i) abdominal obesity: waist circumference ≥90 cm (men) or ≥85 cm (women); (ii) hyperglycemia: fasting plasma glucose ≥6.1 mmol/L or 2-h post-load glucose ≥7.8 mmol/L on oral glucose tolerance test (OGTT), or previously diagnosed diabetes currently under treatment; (iii) elevated blood pressure: systolic blood pressure ≥130 mmHg and/or diastolic blood pressure ≥85 mmHg, or previously diagnosed hypertension currently under treatment; (iv) hypertriglyceridemia: fasting triglycerides ≥1.70 mmol/L; and (v) low high-density lipoprotein cholesterol: fasting HDL-C < 1.04 mmol/L.

Exclusion criteria: (i) medical conditions—severe hepatic or renal impairment, immunodeficiency disorders, active infectious diseases, type 1 diabetes, malignant hypertension, malignancy, thyroid dysfunction, electrolyte disturbances, acute diabetic complications or severe chronic complications (including cardiovascular or cerebrovascular events within the preceding month), prior bariatric surgery, or recent significant weight change (>5% over 3 months or >10% over 6 months); (ii) dietary factors—adherence to specialized therapeutic diets or substantial dietary modification within 12 weeks prior to enrollment; (iii) cognitive and functional status—severe medical or psychiatric disorders, or cognitive, visual, or hearing impairments that would preclude study compliance; (iv) medication and supplement use—current use of omega-3 PUFA supplements or medications known to affect lipid metabolism (concomitant medication use will be systematically documented during the screening visit via a comprehensive medication questionnaire); and (v) allergies—documented egg allergy.

### 2.5 Ethics

This protocol has been reviewed and approved by the Peking University Biomedical Ethics Committee (IRB00001052-25192) and will be conducted in accordance with the ethical principles of the Declaration of Helsinki. Prior to enrollment, eligible participants will be provided with comprehensive information regarding the study purpose, procedures, anticipated benefits, and potential risks. Written informed consent will be obtained from all participants before any study procedures commence. Signed consent forms will be maintained in secure, access-controlled storage.

## 3 Study process

This study is designed as a community-based randomized controlled trial with a 12-week intervention period. Community-dwelling adults aged ≥ 65 years will undergo metabolic syndrome screening, including physical measurements and blood tests, to identify eligible participants based on predefined inclusion and exclusion criteria. A total of 180 participants will be enrolled and randomly allocated into three groups (60 per group) using a computer-generated randomization sequence. The total study duration will be 13 weeks, comprising a one-week baseline assessment period followed by a 12-week intervention. Follow-up evaluations and biospecimen collection will be conducted at weeks 4, 8, and 12 of the intervention, with the final assessment completed at week 13.

### 3.1 Community screening and enrollment

Eligible participants will be identified through screening of physical measurements and blood test data extracted from community health records. Potentially eligible individuals will be contacted by telephone and invited to an in-person information session, during which the study objectives, procedures, benefits, and risks will be explained in detail. Written informed consent will be obtained from all participants prior to any study procedures. Subsequently, trained researchers will administer structured questionnaires to assess baseline dietary intake, medical history, concomitant medications, and nutritional supplement use. Final eligibility will be determined based on strict application of predefined inclusion and exclusion criteria.

### 3.2 Three intervention groups

Omega-3 PUFA-enriched eggs group: Participants will consume one omega-3 PUFA-enriched egg daily, five days per week (Tuesday through Saturday) for 12 weeks. Participants will consume eggs uniformly in the morning to ensure intervention standardization. Monday and Sunday are designated as egg-free days, during which no eggs or egg-derived products are permitted. Eggs will be provided by a centralized supplier along with product quality inspection reports. Nutritional analyses for eggs used in the intervention and control groups will be conducted by the research team.

The omega-3 PUFA-enriched eggs used in this study were tested for nutritional composition by the Chengdu Food Quality Supervision and Inspection Institute in accordance with national standard methods. According to the first method specified in GB 5009.168-2016 (National Food Safety Standard: Determination of Fatty Acids in Foods), the total omega-3 content (sum of α-linolenic acid and docosahexaenoic acid) was 0.446 g per 100 g of eggs, including 0.335 g of α-linolenic acid (ALA) and 0.111 g of docosahexaenoic acid (DHA). Based on the first method of GB 5009.82-2016 (National Food Safety Standard: Determination of Vitamin A, D and E in Foods), the vitamin A content was 201 μg per 100 g and the vitamin E content was 4.41 mg per 100 g. Based on an edible portion of approximately 50 g per egg, each omega-3 PUFA-enriched egg provides approximately 0.223 g of total omega-3 fatty acids. According to the Chinese Dietary Reference Intakes (2023 Edition), the adequate intake (AI) of ALA for adults is 1,600 mg/day for men and 1,100 mg/day for women. The recommended adequate intake of DHA+EPA for adults is 250 mg/day, including 200 mg/day of DHA. Therefore, one omega-3 PUFA-enriched egg per day provides approximately 167.5 mg of ALA, accounting for 15.2% of the AI for women and 10.5% for men, and approximately 55.5 mg of DHA, accounting for 27.8% of the recommended adult intake, which can serve as an important dietary source of omega-3 fatty acids.

Regular eggs group: Participants will consume one regular egg daily, five days per week (Tuesday through Saturday) for 12 weeks. Participants will consume eggs uniformly in the morning to ensure intervention standardization. Eggs will be provided by a centralized supplier along with product quality inspection reports. Nutritional analyses for eggs used in the intervention and control groups will be conducted by the research team.

Blank control group: Participants will maintain their habitual dietary patterns throughout the 12-week study period.

In addition, during the subsequent intervention period, regular testing of omega-3 and other components in each group will be performed to verify and ensure that the intervention dose is clear and reproducible. This study will allow participants to consume other foods containing omega-3 fatty acids, provided that detailed records are maintained in dietary logs. Dietary intake will be assessed using the Food Frequency Questionnaire (FFQ) combined with 3-day 24-h dietary recalls. Participants will be required to provide particularly detailed documentation of omega-3-rich foods. During data analysis, these dietary records will be rigorously analyzed to evaluate their potential impact on the study outcomes.

### 3.3 Universal study requirements and adherence promotion strategies

Consumption of deep-sea fish oil and other omega-3 fatty acid supplements is prohibited for the duration of the trial.

Strategies to enhance intervention adherence will mainly include the following: (1) Ensuring timely supply and accessibility of eggs. Eggs will be delivered weekly to the dining center of the community health service center (CHSC) nearest to the participants. For elderly participants who cannot collect eggs daily from the CHSC, eggs will be mailed or delivered to their homes on a weekly basis. These measures will prevent adherence interruptions caused by delayed delivery of the intervention eggs. (2) Ensuring appropriate cooking methods. Eggs will be boiled or steamed daily at the CHSC dining center, and elderly participants will collect and consume them every morning. For participants receiving home delivery, instructions on boiling or steaming eggs will be included with each delivery, reminding them to use these methods rather than frying or other cooking methods. (3) Targeted reminders and supervision. Each elderly participant will be assigned a researcher responsible for follow-up and reminders. Reminders will be delivered via telephone follow-up (once weekly) combined with smartphone/WeChat messages (daily timed push notifications). Traditional telephone reminders will be retained for those unfamiliar with smart devices. Family members or primary caregivers living with the elderly participants will be invited to participate in “monitor” training to assist with reminders and daily observation. A consumption log will be provided, and participants will check off each date upon consuming one egg, creating a visual completion prompt. (4) Implementing behavioral interventions and incentives. Participants will be instructed to consume eggs at breakfast to leverage the regularity of breakfast habits and improve execution stability. Participants will be provided feedback on changes in relevant biomarker levels (e.g., Omega-3 Index) to enhance motivation through biomarker visualization. Participants will be allowed flexibility in scheduling consumption within a week (e.g., consuming on Thursday if missed on Wednesday), but strict prohibition against consuming ≥2 eggs in a single sitting will be enforced to avoid overconsumption.

For monitoring and assessing intervention adherence, the following methods will be employed: (1) Subjective assessment methods: The 3-day 24-h dietary recall and Food Frequency Questionnaire (FFQ) will be used to record egg consumption timing, quantity, and accompanying foods, with cross-validation between the two methods. Trained interviewers will conduct telephone follow-ups weekly using open-ended questions such as: “What did you eat for your three meals yesterday? Did you consume an egg? At what time?” (2) Objective assessment methods: Fasting venous blood samples will be collected to measure biomarkers of omega-3 fatty acid intake, specifically the Omega-3 Index (the percentage of EPA and DHA in total fatty acids in red blood cell membranes). (3) Process monitoring: Empty egg packaging from the previous week will be collected, and remaining egg counts will be verified against self-reported consumption.

Through this three-tier adherence management system (supply guarantee, reminder supervision, and biological verification), we will maximize the assurance, supervision, and monitoring of stable egg consumption behavior among elderly participants during the 12-week intervention period, thereby enhancing the internal validity of the study.

### 3.4 Outcome indicators and assessment schedule

**Measurement time points:** Baseline (Week 0), and Weeks 4, 8, and 12 of the intervention.

**Baseline week:** Prior to randomization, baseline data will be collected, including general health status, physical activity, sleep quality, mood, cognitive function, dietary intake, and anthropometric measurements. Additionally, fasting antecubital venous blood samples will be obtained to assess glucolipid metabolic parameters.

### 3.5 Intervention initiation and follow-up data collection

Intervention week 0 (first day of Week 2): During the baseline week, structured questionnaires will be administered to assess dietary intake, psychological status, sleep quality, physical activity, cognitive function, medical history, and concomitant medication and supplement use. Trained physicians will perform standardized anthropometric measurements, including height, weight, waist circumference, blood pressure, and body composition. Participants will be instructed to collect midstream urine samples from the first morning void using clean, sterile disposable containers, which will be transferred to pre-labeled urine collection tubes for storage and subsequent analysis. Fresh stool samples will be self-collected in sterile containers and delivered to research personnel for gut microbiota and metabolomic analyses. Fasting antecubital venous blood samples will also be obtained for outcome assessment. Participants will complete and submit three-day dietary records for verification. They will then receive eggs corresponding to their randomized group allocation, along with a “Participant Diary” for daily recording of egg consumption, supplement use, and medication intake. Diary entries and video documentation of egg consumption will be uploaded to a secure online platform to enable real-time monitoring and compliance verification by research staff. Community physicians will evaluate chronic disease complications and intercurrent symptoms based on physiological parameters, with detailed documentation of onset times, treatments, and durations to facilitate subsequent data analysis.

Intervention Weeks 4, 8, and 12: Follow-up assessments will mirror the Week 0 procedures. Fasting antecubital venous blood samples will be collected by trained medical personnel at each visit.

**Figure 1:**
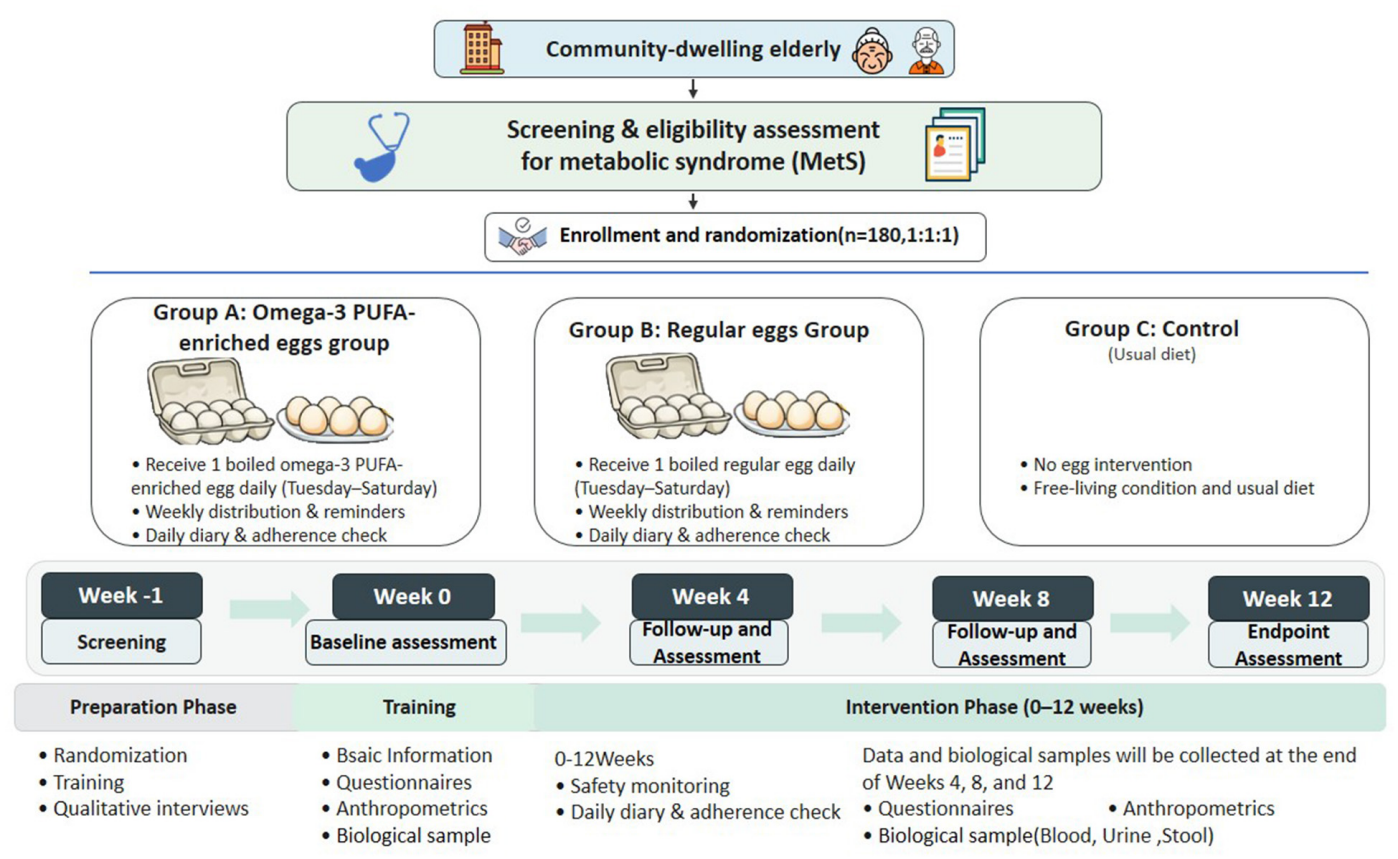
The study flowchart.

Participants who require medical treatment, initiate lipid-lowering therapy, or experience acute illness or exacerbation of chronic conditions during the intervention period will have the onset time, duration, and treatment measures documented in detail. Dietary assessments and other measurements will be scheduled within prespecified time windows for each study phase to minimize potential confounding effects. The study flowchart is presented in Figure 1. The schedule for enrollment, intervention, follow-up, and assessment is shown in Table 2. The intervention components and responsible personnel for the study are summarized in Table 3.

**Table 2:** Time schedule for enrollment, intervention, follow-up, and assessment.

Time point	Enrollment and informed consent	Questionnaire survey	Biological samples collection (blood, urine, stool samples)	Egg consumption and medication/supplement use	Clinical measurements	Outcome evaluation
Enrollment period	√	√		√		
(Baseline week)				√		
The first day of enrollment			√	√		
Intervention phase I (Week 1–4)				√		
First follow-up (end of Week 4)		√	√	√		
Intervention phase II (Week 5–8)				√		
Second follow-up (end of Week 8)		√	√	√		
Intervention phase III (Week 9–12)				√		
Third follow-up (end of Week 12)		√	√			
Laboratory testing phase					√	
Outcome analysis phase						√

**Table 3:** Details of the intervention components and responsible personnel.

Intervention components	Responsible personnel
Group A: omega-3 PUFA-enriched eggs group	
**Dietary intervention (egg consumption):** Tuesday to Saturday: One **boiled omega-3 PUFA-enriched egg daily**; Monday and Sunday: **Egg-free days** (no egg or egg product consumption); Duration: 12 weeks. **Quality control:** Eggs will be distributed weekly, with daily intake recorded. “Verbal + written” reminders will be provided on egg-free days; the diary will be verified and marked for “additional egg/egg product consumption.”	Participants (eggs consumption and diary-keeping) On-site study staff (Distribution of eggs and diaries; compliance monitoring and review)
**Adherence management:** The “Participant Diary” will be collected and reviewed daily. On the first day of each week, the next week's eggs will be distributed, and the previous week's diaries will be collected and reviewed. **Quality Control:** Missing entries will be completed on the same day; anomalies (under-consumption, over-consumption, or non-adherence to egg intake requirements) will be recorded and marked immediately. Compliance will be calculated as “actual consumption/required consumption.”	On-site study staff (Weekly distribution of eggs and collection of diaries) Remote study staff (Daily diary review and quality control)
**Follow-up preparation (Weeks 4, 8, and 12):** Consumables (urine cups, catheters, and stool specimen containers) will be distributed before follow-up visits to remind participants to fast for the next day's physical examination and to collect morning urine and stool samples. **Quality control:** Dual reminders via telephone and in person; consumable ID and distribution registration; verification of sample labels and collection times upon arrival.	**Community physicians** Remote study staff (Daily diary review and quality control)
**Follow-up assessments (Weeks 4, 8, and 12):** Questionnaire administration, physical measurements, blood and biospecimen collection; cold chain transport of samples. **Quality control:** On-site review of questionnaire completeness; defined retest thresholds for physical measurements; end-to-end traceability of samples throughout the “labeling-registration-storage-cold chain” process.	On-site study staff (Weekly distribution of eggs and collection of diaries)
**Safety monitoring:** adverse events (e.g., gastrointestinal discomfort) will be recorded and managed during follow-up visits.. **Quality control:** Severe events will be reported according to established procedures, with discontinuation/withdrawal decisions documented concurrently.	Online surveyors and community physicians
Group B: Regular eggs group	
**Dietary intervention (egg consumption):** Tuesday to Saturday: **One boiled normal fresh egg** daily; Monday and Sunday: Egg-free days; Duration: 12 weeks. **Quality control**: Identical to Group A (consistent frequency and documentation).	Participants (eggs consumption and record-keeping) On-site study staff (Distribution of eggs and diaries; compliance monitoring and review)
**Adherence management/Visit preparation/Follow-up assessments/Safety:** Procedures and quality control measures will be identical to those in Group A (daily diary review, weekly distribution, distribution of consumables and fasting reminders before follow-up at Weeks 4, 8, and 12, sample collection on follow-up days with cold chain transport).	On-site study staff (Distribution of eggs and diaries; compliance monitoring and review)
Group C: Blank control group	
**Usual diet:** Participants will maintain their habitual dietary patterns throughout the 12-week study period. **Quality control:** Potential contamination behaviors, including self-initiated consumption of eggs or egg products will be monitored through diaries and dietary surveys. These data will be used as covariates or for sensitivity analyses.	Participants (record-keeping)
**Follow-up assessments (Weeks 4, 8, and 12):** Follow-up visits and sample collection will be conducted at the same frequency as Groups A and B, including questionnaire administration, physical measurements, and blood, urine, and stool sample collection. All samples will be transported via cold chain. **Quality control:** Quality control procedures will be identical to those in Groups A and B, including on-site questionnaire review, threshold-based review of physical measurements, and end-to-end sample traceability.	On-site study staff (Distribution of eggs and diaries; compliance monitoring and review)
**Data management (all groups):** Data recording, review, and database lock. **Quality control:** Double data entry and review; source data verification for outlier detection.	Statistician

### 3.6 Safety monitoring and adverse events

Systematic safety assessment: Participants will be monitored for gastrointestinal symptoms (e.g., abdominal pain, diarrhea) during follow-up visits at weeks 4, 8, and 12. Liver and kidney function tests will be performed at baseline and at the end of the intervention. The onset time, severity, management, and outcomes of all adverse events will be documented in detail.

Discontinuation criteria: If more than three serious adverse events related to the intervention occur, cases will be submitted to study staff for evaluation, and the trial will be terminated if deemed necessary.

## 4 Randomization and allocation concealment

### 4.1 Random sequence generation

A simple randomization sequence will be generated by an independent statistician using SAS 9.4. The randomization list will be sealed and retained by the statistician in a secure location.

### 4.2 Allocation concealment and implementation

The random sequence will be sealed in sequentially numbered, opaque, tamper-evident envelopes. A non-enrolling investigator will open the envelopes and assign eligible participants to groups, while enrolling investigators will remain blinded to the allocation sequence throughout the recruitment period.

### 4.3 Blinding method

An open-label control with blinded intervention arms design will be adopted in this study. Of the three randomly allocated groups, only the blank control group will be unblinded and aware of their non-intervention status. The control group and the omega-3 PUFA-enriched eggs group will be administered using a blinded protocol, where participants will be informed that they will receive egg intervention but will remain blinded to the specific type (regular fresh eggs vs. omega-3 PUFA-enriched eggs). The two egg types will be identical in appearance and distinguished only by coded labels. Blinded personnel will also include intervention administrators, outcome assessors, and data analysts. Throughout the entire trial, intervention administrators, outcome assessors, and data analysts will be informed only of the unique identification codes of the participants and will be unaware of the intervention types corresponding to each group. The random allocation table will be encrypted and stored by an independent third-party biostatistician, and centralized unblinding will be performed only after the trial has been completed and the data have been locked.

### 4.4 Data collection and quality control

Intervention staff will comprise public health physicians certified in nutrition, and sample collection will be performed by registered nurses with clinical laboratory experience. All personnel will complete standardized training prior to conducting enrollment, follow-up assessments, and biospecimen collection. Instruments and reagents will be calibrated and quality-controlled per SOPs at baseline and at scheduled intervals.

## 5 Study outcomes

### 5.1 Time window for outcome measurement

Outcome assessments will be conducted at baseline (Week 0) and at Weeks 4, 8, and 12. Physical examinations and questionnaire surveys will be completed at each visit. Additionally, serum, urine, and stool samples will be collected for biochemical and multi-omics analyses, enabling longitudinal assessment across pre-, mid-, and post-intervention phases. At each study site, sampling materials will be distributed prior to the Week 4, 8, and 12 visits. Participants will be reminded to fast overnight and to bring first-morning urine and stool samples. On the visit day, physical measurements, blood sampling, and questionnaire administration will be completed. Biospecimens will then be transported under cold-chain conditions to Peking University for subsequent analysis. The time window for outcome measurement is presented in Table 4.

**Table 4:** Time window for outcome measurement.

Outcome		Time point				Sample collection/Method
		Baseline	4 weeks	8 weeks	12 weeks	
**Omega-3 PUFA biological marker**		√	√	√	√	Blood samples
**Glucose metabolism-related parameters**		√	√	√	√	
**Lipid metabolism-related parameters**		√	√	√	√	
**Metabolomics**		√	√	√	√	Blood, urine, and stool samples
**Intestinal flora**		√	√	√	√	Stool samples
**Questionnaire survey**	**Food intake**	√	√	√	√	Food frequency questionnaire (FFQ)/3-day 24-h dietary record
	**Physical activity**	√	√	√	√	International physical activity questionnaire short form (IPAQ-SF)
	**Sleep quality**	√	√	√	√	Pittsburgh sleep quality index (PSQI)
	**Mood state scale**	√	√	√	√	Profile of mood states (POMS)
	**Cognitive scale**	√	√	√	√	Montreal cognitive assessment (MoCA)
**Physical measurements**		√	√	√	√	Stadiometer, weight scale, flexible measuring rule, bioelectrical impedance analyzer

### 5.2 Plasma omega-3 PUFA as biomarkers

In this study, plasma omega-3 PUFAs will serve as objective biomarkers of intervention exposure, reflecting changes in participants' omega-3 intake and absorption, and enabling assessment of their relationship with metabolic outcomes. Samples will be collected at baseline (Week 0) and at Weeks 4, 8, and 12 of the intervention, coinciding with primary follow-up visits. Fasting venous blood samples will be collected, and plasma will be separated and stored at −80 C until analysis. Plasma concentrations of DHA, EPA and ALA will be quantified. Results will be expressed as absolute concentrations and, if necessary, as percentages of total fatty acids to facilitate inter-individual comparisons (22). Total lipids will be extracted from plasma using standardized procedures and quantified by gas chromatography (GC-FID or GC-MS) or liquid chromatography-tandem mass spectrometry (LC-MS/MS). Internal standards and quality control samples will be included in each assay batch to ensure consistency (23). These measurements will validate enhanced *in vivo* omega-3 exposure in the omega-3 PUFA-enriched egg group relative to the standard egg group, supporting subsequent pathway analyses of the “omega-3 exposure (DHA/EPA/ALA changes)–metabolic phenotype (e.g., TG)–omics signatures” relationship (24, 25).

### 5.3 Primary outcome

The primary outcome will be defined as the longitudinal trajectory of fasting triglycerides (TG) measured at Weeks 0, 4, 8, and 12. The primary statistical analysis will focus on the group × time interaction, indicating differences in TG trends over time among intervention groups. The pre-specified primary comparison will be between the omega-3 PUFA-enriched egg group and the standard egg group to evaluate intervention efficacy. Exploratory pairwise comparisons will also be conducted for other group combinations not specified in the primary hypothesis.

### 5.4 Key secondary outcomes and exploratory outcomes

Blood samples will be collected at baseline (Week 0) and Weeks 4, 8, and 12 to assess indicators related to glucose and lipid metabolism (four samples per participant, covering lipid profiles, glucose homeostasis, inflammatory markers, oxidative stress, and other metabolic parameters). Samples will be collected following an overnight fast, with serum and plasma separated according to standardized protocols. Aliquots will be stored at −80 C and shipped in batches for centralized analysis. Biochemical and immunological markers will be quantified using enzyme-linked immunosorbent assay (ELISA) and other validated methods, with rigorous technical support to ensure intra- and inter-assay consistency and full traceability.

Key secondary outcomes:

(1) Lipid metabolism indicators included total cholesterol (TC), triglycerides (TG), high-density lipoprotein cholesterol (HDL-C), low-density lipoprotein cholesterol (LDL-C), free fatty acids (FFA), lipoprotein(a) [Lp(a)], apolipoprotein A1 (ApoA1), and apolipoprotein B (ApoB). These indicators will provide a comprehensive assessment of lipid metabolism disorders and associated cardiovascular risk.
(2) Inflammation and oxidative stress markers: Measured indicators will include high-sensitivity C-reactive protein (hs-CRP), interleukin-1 (IL-1), interleukin-6 (IL-6), tumor necrosis factor-alpha (TNF-α), and oxidative stress markers such as superoxide dismutase (SOD) and glutathione (GSH).

Exploratory outcomes:

(1) Glucose metabolism and insulin resistance indicators: Measured markers will include fasting plasma glucose (FPG), glycated hemoglobin (HbA1c), fasting insulin, and C-peptide to assess glycemic control and pancreatic β-cell function. Additionally, the homeostasis model assessment of insulin resistance (HOMA-IR) will be calculated as: HOMA-IR = [FPG (mmol/L) × FINS (mU/L)] ÷ 22.5, where higher values indicate greater insulin resistance.
(2) Metabolomics: This study will employ untargeted metabolomics to characterize the systemic effects of omega-3 PUFA-enriched egg intervention on metabolic phenotypes, aiming to identify potential metabolic pathways and biomarkers associated with primary clinical outcomes. Plasma, urine, and stool samples will be collected at baseline (Week 0) and follow-up visits (Weeks 4, 8, and 12), then stored at −80 C pending analysis. Metabolite features will be detected using liquid chromatography-mass spectrometry (LC-MS). To ensure reproducibility, standardized protocols adhering to the Minimum Reporting Standards for Metabolomics and Chemical Analysis Reporting Elements will be implemented throughout sample preparation, instrument operation, metabolite annotation, identification, and data preprocessing (26). Additionally, quality control samples will be incorporated to monitor analytical drift and batch effects, with corrections applied as needed to enhance data quality and comparability (27). Metabolomic results will provide exploratory mechanistic insights, which will be interpreted in conjunction with clinical phenotypes and findings from other omics analyses.
(3) Gut microbiomics: Gut microbiota-related indicators will be evaluated as exploratory mechanistic outcomes to elucidate potential gut-derived mechanisms underlying metabolic phenotype changes (e.g., triglycerides) in older adults with metabolic syndrome following omega-3 PUFA-enriched egg intervention. Primary microbiome measures will include α-diversity, β-diversity, community structure comparisons, and analyses of differential microbial abundance and functional pathways.

Stool samples will be collected at baseline (Week 0) and at Weeks 4, 8, and 12, and then stored at−80 C for subsequent analysis. The primary analytical endpoint will align with the clinical outcomes (Week 12), while samples collected at Weeks 4 and 8 will be analyzed to explore temporal changes and potential mechanisms. To ensure reproducibility and comparability, all procedures—including sample collection, storage, DNA extraction, sequencing, and statistical analyses—will follow standard operating procedures and reporting guidelines for human stool sample processing (28, 29).

### 5.5 Questionnaire data collection

### 5.6 Dietary omega-3 PUFA intake

Dietary omega-3 PUFA intake will be estimated using a semi-quantitative food frequency questionnaire (FFQ), with participants recording food types, intake quantities, and portion sizes using food photograph models (30). Trained researchers will assess dietary omega-3 PUFA intake based on the Chinese Food Composition Table (31).

Participants will also complete detailed dietary records for three consecutive days (including two weekdays and one weekend day) using a 3-day, 24-hour dietary recall form. Food portions will be weighed using calibrated scales (precision 0.1 g). Participants will record the time, type, cooking method, and weight of all consumed foods, with daily records documented through photographs and submitted to research staff for daily review to ensure data accuracy and completeness.

### 5.7 Physical activity

Physical activity will be assessed using the validated international physical activity questionnaire short form (IPAQ-SF). The IPAQ-SF is a self- or interviewer-administered instrument measuring the frequency (days per week) and duration (minutes per day) of walking, moderate-intensity, and vigorous-intensity activities across daily life contexts, and includes a sitting-time item to obtain both continuous and categorical physical activity data (32, 33). Participants will be categorized into low-, moderate-, and high-intensity physical activity groups according to official IPAQ scoring guidelines. To ensure data quality, appropriate data cleaning and truncation procedures (e.g., addressing unusually high durations) will be performed following established IPAQ recommendations, with methods transparently reported in statistical analysis (34). Given the older adult population in this study, previous reliability and criterion-related validation of the Chinese version of IPAQ (IPAQ-C) among Chinese older adults, using pedometer steps as a reference, supports its suitability for this population (35). Wearable devices will not be used to monitor physical activity.

### 5.8 Sleep quality and duration

The pittsburgh sleep quality index (PSQI) will be used to assess sleep quality and duration among participants. The PSQI comprises 19 self-rated items and 5 observer-rated items (36, 37). A total score will be derived from the 19 self-reported items across seven domains: subjective sleep quality, sleep latency, sleep duration, habitual sleep efficiency, sleep disturbances, use of sleep medications, and daytime dysfunction. Higher total scores will indicate poorer sleep quality.

### 5.9 Mood state scale

The Chinese version of the profile of mood states—abbreviated form (POMS-A) will be used to assess participants' mood states over the past week (including the current day). This 40-item self-administered scale comprises seven dimensions: tension, depression, anger, vigor, fatigue, confusion, and self-esteem. Participants will rate 40 affective adjectives on a 5-point Likert scale (0–4). Dimension scores will be summed; except for the positive dimensions (vigor and self-esteem), higher scores will indicate more negative emotional experiences (38–40).

The total mood disturbance (TMD) index will also be calculated to reflect overall emotional distress using the formula: TMD = (Tension + Anger + Fatigue + Depression + Confusion) – (Vigor + Self-esteem) + 100, with higher TMD scores indicating greater negative mood disturbance (41). The Chinese version of the POMS-A has demonstrated good internal consistency and usability in diverse populations, including older adults (38, 42).

### 5.10 Cognitive assessment

This study will use the Chinese version of the montreal cognitive assessment (MoCA) to screen and quantitatively evaluate the overall cognitive function of participants. The MoCA is a rapid, pencil-and-paper screening tool that can be completed in approximately 10 min, with a maximum score of 30 points. It assesses multiple cognitive domains, including visuospatial/executive functions, naming, attention and calculation, language, abstraction, delayed recall, and orientation; higher scores indicate better cognitive function. The original validation of the MoCA recommended a cutoff score of 26 for identifying mild cognitive impairment (MCI) (43). Given that MoCA cutoff thresholds vary across cultural and demographic contexts—particularly among elderly populations with significant educational disparities—a localized validation of the Beijing version of the MoCA has been conducted in a Chinese community study (44). Recent systematic reviews and meta-analyses have shown that there is no fixed optimal MoCA cutoff for the Chinese elderly population; instead, the cutoff should be determined based on the study's objectives and the demographic characteristics (e.g., age, education level) of the participants (45). Therefore, in this study, MoCA scores will be treated as continuous variables, and potential confounding factors such as years of education and age will be adjusted for to ensure the robustness of the analysis.

### 5.11 Physical measurements

At each study visit, trained research personnel will perform standardized anthropometric and blood pressure measurements, with results recorded on physical examination forms. All measurement devices will be calibrated and subjected to quality control procedures prior to use.

Height and Weight: Height will be measured to the nearest 0.1 cm using a wall-mounted stadiometer, with participants standing erect, barefoot, and without headwear. Weight will be measured to the nearest 0.1 kg using a calibrated digital scale, with participants wearing light clothing and no shoes. Each measurement will be performed twice, and the mean value will be recorded. If the difference between two measurements exceeds the predefined threshold, the device will be recalibrated and the measurement repeated.

Waist Circumference: Waist circumference will be measured to the nearest 0.1 cm using a non-stretchable measuring tape at the horizontal midpoint between the lower margin of the costal arch and the iliac crest. The measurement will be performed twice, and the mean value will be recorded.

Blood Pressure: Blood pressure will be measured using a validated automated sphygmomanometer or standard mercury sphygmomanometer (range 0–300 mmHg, accuracy ±2 mmHg). Systolic and diastolic pressures will be determined by Korotkoff sounds. Three measurements will be taken on the left arm, with the cuff fully deflated and a 30-s rest interval between measurements. Participants will remain seated quietly, with no speaking or movement during measurement. The mean of the three readings will be used for analysis.

Body Composition: Body composition will be assessed using a bioelectrical impedance analyzer. Participants will stand barefoot on the foot electrodes and grasp the hand electrodes with both hands, with thumbs lightly contacting the sensors, while maintaining a stationary position for approximately 60 s. Body composition reports will be archived as source documents.

## 6 Confidentiality and withdrawal

All participants' personal information will be kept strictly confidential throughout the study. Unique identification codes will be assigned to each participant to manage their data during and after the trial, and personal identifiers will be stored separately from study data to ensure data security. Paper-based study materials will be stored in locked cabinets, and electronic data will be stored on encrypted servers with restricted access to authorized personnel only. Participants may voluntarily withdraw from the trial at any time without any penalty. Any adverse events that occur during the trial will be managed by emergency physicians to ensure participant safety.

## 7 Data entry and analysis

**Data entry:** Questionnaire data will be collected directly via electronic forms with built-in logic checks to ensure the accuracy and completeness of data entry, minimizing errors in data recording.

**Statistical analysis:** Statistical analyses will be performed using SPSS 24.0 and R 4.2.0 software. For baseline variables, continuous variables will be analyzed using one-way analysis of variance (ANOVA) or kruskal-wallis tests, while categorical variables will be analyzed using chi-square tests or Fisher's exact tests.

For longitudinal outcome measures, mixed-model repeated measures analysis of variance (MMRM) with an unstructured covariance matrix will be adopted as the primary analytical method. This model will include intervention group, time point, and group × time interaction as fixed effects, with subject-specific random intercepts. Pre-specified covariates (including age, sex, body mass index (BMI), baseline dietary intake, physical activity, baseline clinical parameters, and compliance) will be adjusted in the primary analysis based on their correlation with outcomes and clinical relevance.

Missing data will be handled using intention-to-treat (ITT) analysis with mixed models, which can accommodate unbalanced data under the missing at random (MAR) assumption. Sensitivity analyses will include complete case analysis and multiple imputation by chained equations (MICE) to verify the robustness of the results.

For multi-omics analyses (microbiome and metabolomics), Benjamini-hochberg false discovery rate (FDR) correction (*q* < 0.05) will be applied to control the expected proportion of false positives among the results. Microbiome data will undergo centered log-ratio (CLR) transformation to address compositional data constraints. Non-parametric methods [Friedman test, permutation-based multivariate analysis of variance (MANOVA)] will be used when the assumptions of normality or homogeneity of variance are violated. Principal coordinate analysis (PCoA) based on bray-curtis distances will be used to analyze microbiome composition, and principal component analysis (PCA) will be applied to metabolomics data. Associations between the microbiome and metabolome will be analyzed using sparse partial least squares (sPLS) or canonical correlation analysis (CCA), with FDR correction applied to ensure the reliability of the results.

## 8 Discussion

MetS is a common chronic condition among elderly individuals, characterized by disorders in glucose and lipid metabolism, with chronic inflammation as core pathological features. Although omega-3 PUFA effectively regulate lipid metabolism and suppress inflammation, compliance with traditional omega-3 supplementation methods remains generally low among Chinese populations due to insufficient dietary intake. Omega-3 PUFA-enriched eggs provide a convenient dietary intervention approach; however, their effects on elderly individuals with MetS require further investigation. This study design a three-arm, parallel-group randomized controlled trial to evaluate the effects of omega-3 PUFA-enriched eggs on metabolic phenotypes in elderly patients with MetS in a real-world community setting. A 12-week intervention with multiple follow-up assessments will be conducted to systematically explore the practical application and efficacy of omega-3 PUFA-enriched eggs in this population.

This study will incorporate three intervention groups (omega-3 PUFA-enriched eggs, regular eggs, and blank control group) to establish a robust multi-level comparative framework. The comparison between the omega-3 PUFA-enriched eggs group and the regular eggs group will specifically isolate the incremental effect of omega-3 enrichment. The regular eggs and blank control groups will provide references to quantify the basic effects of egg consumption. Direct comparisons between the omega-3 PUFA-enriched eggs and blank control groups will be helpful for revealing the integrated intervention effects. Repeated measurements and biological sampling will be conducted at Weeks 0, 4, 8, and 12, balancing endpoint assessment with dynamic tracking of intervention effects and providing crucial temporal insights into the association between compliance and efficacy. This approach will overcome the limitations of single-endpoint evaluations in capturing intervention dynamics. In addition to standard clinical outcomes, this study will include analyses of multiple biological samples (blood, urine, stool), as well as evaluations of gut microbiota and metabolomics, enabling comprehensive exploration of the underlying biological mechanisms alongside efficacy assessments.

The primary challenges of this study will be compliance management and bias control. To enhance the implementation of the intervention, semi-structured interviews focusing on compliance barriers, intervention feasibility, and facilitating factors will be incorporated during the design phase to ensure alignment between the intervention format and the daily habits of elderly participants. Insights gained from these interviews will improve the feasibility of the intervention and participant compliance by optimizing trial procedures. This integration of qualitative methods will not only facilitate the smooth implementation of the trial but also provide practical guidance for future community-based applications. At the intervention level, rigorous compliance management will be achieved by first establishing trust with participants through clear explanations of the study objectives and significance. Efficient communication channels will be maintained via telephone, WeChat, SMS, and home visits. During the intervention period, follow-up will be conducted using a strategy of “daily participant diary + daily online review by investigators + weekly offline egg distribution and diary collection”.

Regarding data quality control, all researchers will receive standardized training on trial procedures, questionnaire administration, and technical operations. Questionnaires will be collected and reviewed on-site during follow-up visits. Dietary records will be carefully verified, and a double data-entry system with cross-verification will be implemented to ensure high data integrity. Anthropometric measurements—including height, weight, waist circumference, and blood pressure—will be taken twice to enhance accuracy. Food photo models will be used to guide participants in completing dietary frequency questionnaires, thereby improving the accuracy of intake estimation. Participants will receive clear instructions and training on questionnaire completion. Data from participants who no longer meet the study criteria will be excluded, and the reasons for withdrawal will be documented in detail.

For bias control, randomization sequences will be generated by independent statisticians, and allocation concealment will be ensured using opaque envelopes. Group assignment will be performed by non-enrolling investigators to minimize investigator bias. Regarding blinding, this trial will adopt an “open-label control with blinded intervention arms” design. The two types of eggs (omega-3 enriched vs. regular) will be identical in appearance and differentiated only by coded labels. Although participant blinding will not be feasible in the blank control group, the limitations of blinding will manifest in the following aspects. First, the open-label status of the blank control group will make participants explicitly aware that they are receiving no intervention, potentially inducing negative expectation effects and feelings of deprivation, which may lead to deterioration in health behaviors or reporting bias in symptom assessment. Although participants in the two blinded egg groups will be unaware of the specific egg type they receive, being informed of “receiving an egg intervention” may still generate positive expectations. The gradient distribution of expectation intensity across the three groups (hypothesized as: blank control < regular eggs < omega-3 PUFA-enriched eggs) will create an imbalance in expectation effects, making it impossible to distinguish whether between-group differences originate from the intervention itself or from differential psychological expectations. Second, participants in the control groups may voluntarily modify their dietary behaviors (e.g., increasing egg consumption) to compensate for the sense of deprivation, resulting in inter-group contamination and dilution of intervention effects. Third, despite the blinding of intervention administrators, outcome assessors, and data analysts, subjective outcome measures will remain susceptible to participant expectations; while objective biochemical indicators, although relatively robust, could still be confounded by behavioral changes in the control groups. Furthermore, divergent expectation levels among the three groups may lead to heterogeneous variance in outcome variables, and the potentially higher attrition rate in the blank control group could cause non-random missing data, threatening the validity of statistical inference. These limitations will exert multidimensional impacts on internal validity. First, expectation effects cannot be excluded as a confounding factor, meaning that the observed effects of omega-3 PUFA-enriched eggs may represent a combination of true biological effects, placebo effects, and negative expectation effects from the control group, with the weighting of each component remaining indeterminate. Second, it will limit the generalizability of the results to scenarios with uniform blinding or open-label conditions. In conclusion, the open-label status of the control group will constitute a source of systematic bias. Causal language should be used cautiously when interpreting results; priority should be given to objective biochemical indicators, and sensitivity analyses alongside process evaluations will be conducted to quantify the extent of bias. Nevertheless, allocation concealment, third-party randomization, assessor blinding, and post-data-lock unblinding will also be implemented to partially attenuate these biases.

Several other limitations should be acknowledged. First, complete participant blinding will not be achievable due to the absence of egg distribution in the blank control group, which may introduce expectation or behavioral bias. Second, dietary intake and compliance will be partly self-reported, potentially leading to recall or social desirability bias. Such bias will be minimized through strict quality control, double data entry, and validation using objective indicators. Third, the 12-week intervention period in this study will be sufficient to evaluate the acute effects of omega-3 PUFA-enriched eggs on blood lipids, gut microbiota, and metabolomic profiles; however, considering the ecological resilience of gut microbiota, longer intervention durations or post-intervention follow-up will be beneficial for assessing the sustainability of these changes. Future research will explore 6-month intervention designs with washout periods to verify the stability of gut microbiota alterations. Finally, variability in cooking methods and dietary substitution may influence individual exposure levels and metabolic outcomes. Although boiled eggs will be provided to participants living near community hospitals, cooking practices cannot be fully controlled in elderly individuals with limited mobility. Therefore, dietary surveys and follow-up records will be incorporated for covariate adjustment and sensitivity analyses, enhancing the robustness of result interpretation.

Regarding the innovation of this study, the research focuses on eggs as a delivery vehicle for omega-3 fatty acids. While existing omega-3 intervention studies have primarily relied on fish oil or algal oil capsule supplements— which have demonstrated benefits for gut microbiota and metabolic health—elderly populations face significant implementation barriers, including dysphagia, gastrointestinal intolerance, and poor long-term adherence. This study will employ omega-3 PUFA-enriched eggs as a daily food-based delivery vehicle, which combines high bioavailability with dietary accessibility, providing a sustainable and scalable alternative for elderly adults with metabolic syndrome. Research on this specific delivery vehicle remains limited. Regarding the target population, this study will focus on elderly adults with metabolic syndrome. Although the multi-omics mechanisms of omega-3 PUFAs in metabolic health have been extensively explored, integrated intervention studies specifically targeting elderly MetS patients remain scarce. Elderly adults exhibit unique metabolic characteristics (sarcopenia, insulin resistance, decreased gut microbiota diversity) and intervention sensitivity; this study will address the evidence gap for food-based omega-3 PUFA-enriched egg interventions in this specific subpopulation. Methodologically, this study will integrate plasma omega-3 exposure biomarkers, clinical metabolic phenotypes, and multi-omics endpoints to establish a longitudinal “exposure-microbiota-metabolite-phenotype” association network. Distinguishing from traditional designs that analyze clinical or omics endpoints in isolation, this study will employ mixed-model repeated measures analysis to reveal dynamic response patterns, providing an integrative perspective for understanding the systemic mechanisms of omega-3 PUFA-enriched eggs on health outcomes. Additionally, this study will concurrently assess adherence and acceptability during the 12-week intervention, documenting the dose-response relationship between actual egg consumption and plasma omega-3 responses, thereby providing implementation science evidence for the development of community-based nutritional intervention strategies for elderly adults.

In conclusion, this study will aim to evaluate the intervention effects of omega-3 PUFA-enriched eggs in patients with MetS, while systematically exploring the underlying mechanisms. The findings will provide valuable scientific evidence for related research and practical applications in community-based nutritional interventions for the elderly.
